# The Danger to the Community of the "Beauty Specialist"

**Published:** 1909-10-23

**Authors:** Morgan Dockrell

**Affiliations:** Senior Physician to St. John's Hospital for Diseases of the Skin, and Chesterfield Lecturer


### SPECIAL ARTICLE.

### THE DANGER TO THE COMMUNITY OF THE "BEAUTY SPECIALIST."

By MORGAN DOCKRELL, M.D., Senior Physician to St. John's Hospital for Diseases of the Skin, and Chesterfield Lecturer.

(Opening Lecture delivered at the Hospital on October 7, 1909.)

One of the significant characteristics of all highly civilised peoples has been the large amount of time and attention they have devoted to the cultivation of personal beauty of form and feature.

In ancient Greece and Rome men, equally with women, gave up a by no means small portion of their daily lives to this end. For, as a matter of fact, their luxurious system of baths, public and private, their long and arduous training in all forms of games and athletics were primarily but adjuncts and aids to developing, refining, and beautifying the human form.

Now, ladies and gentlemen, just as those peoples in their generations were wiser and more highly intuitive than are the moderns as regards some of the fine arts and literature, so in this matter of the development and preservation of physical beauty they had a much firmer grasp of the true value and meaning of such beauty. For they had a keen apprehension of the fact that physical beauty is dependent on, and entirely the outward and visible sign of, health.

Taking our modern civilisation in bulk, it may very safely be asserted that there is no such keen or universal apprehension of this fact. Among men there is a good deal of emphasis laid on the all-importance of keeping fit, but side by side with that, an utter disregard of the elementary hygienic essentials conducive to fitness. So you often find your city man in search of fitness going country journeys, traversing long miles of golf links, all for health and health's sake; yet spending his indoor life for the most part in an atmosphere reeking with impurities and saturated with the germs of disease. The simple antidote of thorough ventilation he either neglects or objects to, and consequently golf and never-ending change of air and scene notwithstanding he neither is, nor feels, nor looks fit. Among women there is, alongside a never-increasing expenditure on the attempt to preserve and enhance the charms bestowed on them by Nature, an appalling amount of ignorance of the elementary rules of hygiene relative to pure air, wholesome food, sufficiency of exercise and rest, and the right observation of strict cleanliness in the widest sense and amplification of the word, all of which are the very first essentials on which beauty depends. Their neglect brings the usual Nemesis, the dreaded wrinkles that label the flabby muscles, the fading complexion, the sallow, the spotty or the coarsened skin. Consequently there is a dangerously increasing tendency to fly for remedy, *not to Nature and natural processes*, but to artifice and artificial measures. Meanwhile what may be summed up as Charlatan Æstheticism thrives on the credulity of the foolish crowd, who flock like flies round a honey pot to the latest quack who promises beauty of form and feature and the restoration of the bloom of youth out of her grease pots and mechanical appliances.

With the numerous artificial means and methods to which women are submitting their bodies in order to be beautiful, and which very often result in shattered health and nervous breakdown, we are only concerned here this evening in so far as they are applied to the department of medicine coming under the head of Dermatology, and before going any farther I want to emphasise these two facts. I am neither making an attack on many of those persons who are legitimately, I believe, earning a living by carrying out various harmless toilette processes, which, as in the case of properly administered face massage, do no doubt aid in the preservation of good looks, and the prevention of lines and wrinkles. Nor am I condemning the judicious use of cosmetics. While quite convinced that the too prevalent use of face powders and greases, absurdly misnamed skin foods, is responsible for—in the one case many prematurely withered, and in the other relaxed and flabby necks and faces, I know there are occasions when an irritated or inflamed face is soothed and beautified by the application of a pure powder or lotion.

My tilt this evening is against a different class and different methods. These beauty doctors, so-called, utterly ignorant of anatomy or physiology, do not hesitate to undertake the treatment and cure of many cases where the symptoms to the uneducated eye may be but a slight blemish or a small unsightly stain. They look on the human skin very much from the standpoint of a tailor or dressmaker planning the trimming or alteration of a garment. They approach it as if it were a totally independent organ. Now, as we know—and the more fully we apply the knowledge in our treatment of skin diseases so much the more shall we be successful in our treatment—the skin and the general health of the system are always acting and reacting one on the other, and there is no possibility of anything even approximating to bodily health if the skin be diseased. Just as we know that the apparently trifling blemish, the slight stain, the tiny nodule, the colour and texture of the skin, which convey nothing to the uninitiated, will speak volumes to the practised eye of the medical man and give him at a glance an inkling of quiescent or threatening grave disease. There are numerous cases of long time suffering and permanent disfigurement where both pain and disfigurement might have been altogether obviated but for the tinkering interference of these social pests. We are all familiar with the muddied complexion of seborrhœa. Take one such case as an example of what happens when the beauty shop-woman undertakes a cure. Of course, the treatment varies in ignorance at different shops. One very general treatment is to sell the patient a very expensive and highly irritating soap, and a lotion. Soap and lotion are to be used twice daily. After a few days extensive scaling takes place, followed not seldom by acute eczema. For this stage the so-called soothing treatment is adopted; more lotions and ointments—needless to say all expensive—are sold. After a few weeks of this treatment the patient, if wise, seeks medical advice, when it is found that she is suffering from either chronic eczema, with its accompanying disfiguring lines; or, should the treatment have been with sulphur lotion, followed by a mercurial ointment, the face is covered by what appears to be blackheads. So that you see, ladies and gentlemen, the last state of such a case is infinitely worse than the first.

In another establishment the treatment consists in covering the face with towels wrung out of boiling water, a mode of beautifying now being largely employed, too, in many men's hair-dressing saloons, chiefly patronised by silly boys and more silly old men. Over these hot towels there is vigorous application of a vibrator for ten to fifteen minutes. A highly spirituous lotion is afterwards applied. The results are: For a few succeeding hours a pink and white complexion; for days, or perhaps weeks, facial neuralgia, erysipelas at times, and acute eczema; and in two cases lately coming under my notice ul-erythema, that incorrigible disease formerly known as lupus erythematosus.

Again, take those cases where papules or spots occur on the face. These, of course, constitute the beauty specialist's harvest field. It is here she employs her remedies with a free hand. A spot to her is a spot.

Now let us consider for a few minutes some of those cases where some apparently trifling blemishes of the skin may be but symptoms of grave disease. Take, for instance, a few of the various forms of papules commonly summed up by the beauty-doctor under the head of spots or blotches.

There are the different forms of acne, of tubercular nodules, and of syphilitic papules. In each of these the papule or nodule is merely a danger signal indicating its own accompanying form of disease, and, of course, requiring its peculiar form and method of treatment.

A patient consults the beauty-doctor, say, for that form of acne commonly called blackheads. The following is the common form of treatment adopted: The blackheads are squeezed out. Not seldom the forcible expulsion sets up considerable inflammation, leaving behind a little scar, presently to be followed by a minute hole. Now unless at this stage there is proper treatment, this small hole may, and probably will be, invaded by one of the staphylococci, setting up pus, resulting in a large scar irregular in shape and due to loss of tissue. This is bad enough, but you will realise how far more serious may be the consequences when this scarring takes place on a tuberculous soil. Then the patient suffers all the pain and soreness of deep scarring and a pitiable state of deformity of features.

Another method is, after forcible expulsion of the blackheads, the use of the vaporiser and application of lotions. The use of these lotions, some of them misnamed stimulating, but in reality highly irritating, in certain conditions of the skin sets up an inflammation which causes an entire skinning of the face, or results in such a constitutional modification of the papules that instead of scarring and small holes, there may be deep-seated scars with complete destruction of the sebaceous glands, those necessary sources from which the skin derives its softness and pliability.

Now we come to tuberculosis of the skin, commonly known as lupus. The patient perhaps has a nodule, or tiny groups of nodules, on the face, which are described to her by the quack as warts, and as very easily removable under treatment. These nodules are soft, easily broken down and liable to ulceration if subjected in any way to irritation. Others are the papules often seen in those who have undergone treatment for consumption in Sanatoria. An attempt is made to remove the so-called wart by either the use of acids or by electrolysis. What follows? There is an existing, but probably quiescent, tuberculosis of the skin. The irritation arouses and aggravates the disease, with the result in some cases that chronic trouble is set up, which requires incessant watching and treatment to keep in check. Or where the irritation has not taken such form it has been followed by enlargement of the glands. I have had several cases with this history within the last few years.

If the nodule is, as sometimes occurs, on the side of the nose, it is no infrequent thing for ulceration to ensue, which leaves a small hole caused by a piece coming away from the edge of the nostril.

Now if the diagnosis had been made in these cases and there had been no local irritation, there would have been no small hole made, no ulceration, no piece coming away at the edge of the nostril. The whole condition with constitutional treatment would have subsided without scar or blemish, leaving no trace behind.

In cases where some facial blemish is due to hereditary or acquired syphilis the unscientific treatment is rapidly followed by the very gravest consequences. After a few daubings with the beauty-doctor's mixtures the disease assumes a condition that is alarming even to the uninitiated and inexperienced eye, and the subsequent treatment undergone after seeking medical advice is long and wearisome, and in some cases there can be no obliteration of the marks left by scarring.

In some of these cases of unsuspected hereditary syphilitic taint the disastrous element in the case is that had a medical man been consulted in the first instance about the small supposed disfiguring wart, a correct diagnosis would have been arrived at, constitutional treatment would have been undergone, and much subsequent misery and danger obviated.

Red face and superfluous hair on the face are the sheet anchor of the beauty doctor. Electrolysis is for both their favourite *modus operandi.* Indeed, so common is the use of electrolysis, that anyone may set up as an operator. And yet, ladies and gentlemen, there is no more frequent source of disfigurement owing to a bungling or careless operator and want of knowledge as to even the proper pole with which to apply the needle, the least serious consequences of which are the tiny scars often seen on women's upper lip and chin, or the permanent stain resulting from unskilled operating.

The more serious are where its misuse, as in the case of the condition called red face, only aggravates the trouble. So that sometimes a woman whose face is merely temporarily inflamed or irritated from exposure, say, after a season's yachting or a motor tour, as a result of electrolysis is affected with the disfigurement in its chronic form.

The nævi of telangiectasis, congenital or acquired —contribute to the red face, and invariably occur in persons with very thin skins. The use of acids and electrolysis in such cases is directly opposed to indications, as the skin is so much more liable to irritation and scarring.

And now, ladies and gentlemen, having touched very briefly on this subject as to the danger arising from unqualified and ignorant treatment of diseases of the skin, I should like to add a word about it from another point of view.

Very frequently, in cases where there is long-continuing noticeable disfigurement, the patient suffers a pitiable amount of distress of mind, going on at times to nervous breakdown. The reason so few of these cases are heard about by the general public is that there is almost invariably on the part of the patient and her relatives alike a very natural dread of publicity. Few and far between are those who will consent to take part in any exposure of this nefarious trade and those who practise and thrive on it. In fact, the sufferer's dread of publicity is the quack's safeguard and protection. Now and then at long intervals some Madame Rachel of her day or period, with her methods and poisonous washes and inunctions, is brought into a court of law. But in 99 cases out of 100 the patient and her relatives prefer to have the matter hushed up. Not alone so, but it is only with difficulty sometimes that the true history of her case can be got from the patient by the medical man to whom she at length goes for treatment.

Meanwhile the streets of our cities are honeycombed with beauty shops, and the advertisement columns of the daily press crowded with advertisements promising the bestowal of the beauty of Venus on ugliness, and the bloom of youth on age. All of which would no doubt be but food for mirth were it not of such serious import. There is no doubt that sooner or later the State will have to inflict serious penalties on this unqualified treatment of disease, just as more stringent precautions should be taken to prevent the use of dangerous hair washes and shampoos.

And finally, ladies and gentlemen, as a medical man speaking to medical men and women, I think there should be amongst us a much wider realisation of this disheartening fact, that at a time when the medical practitioner, after all his or her years of expensive and arduous scientific training, is finding it in some cases almost impossible to make a decent living, these harpies are allowed to treat diseases and batten on those many gullible people who consult them. Of course, this cult of the beauty doctor is all part and one with the almost universal tendency amongst women to dose themselves with patent medicines. There is to-day an indiscriminating use of drugs that should only be used when prescribed that is little short of suicidal, with the result that there was, I should think, never a time when nervous derangement and nervous breakdown were more alarmingly in evidence. The old-time safeguard of consulting the family practitioner at the beginning of illness or disease and averting grave and often fatal issues has given way to a haphazard trickery and pottering with advertised cures and quackery eventually responsible for a very large portion of the gloom, depression, and pessimism that hangs like a cloud over our modern civilisation.

